# Impact of 5'-amp-activated Protein Kinase on Male Gonad and Spermatozoa Functions

**DOI:** 10.3389/fcell.2017.00025

**Published:** 2017-03-23

**Authors:** Thi Mong Diep Nguyen

**Affiliations:** ^1^Physiologie de la Reproduction et des Comportements, INRANouzilly, France; ^2^Quy Nhon UniversityQuy Nhon, Vietnam

**Keywords:** AMPK, energetic metabolism, spermatozoa, male gonad, fertility

## Abstract

As we already know, the male reproductive system requires less energetic investment than the female one. Nevertheless, energy balance is an important feature for spermatozoa production in the testis and for spermatozoa properties after ejaculation. The 5'-AMP-activated protein kinase, AMPK, is a sensor of cell energy, that regulates many metabolic pathways and that has been recently shown to control spermatozoa quality and functions. It is indeed involved in the regulation of spermatozoa quality through its action on the proliferation of testicular somatic cells (Sertoli and Leydig), on spermatozoa motility and acrosome reaction. It also favors spermatozoa quality through the management of lipid peroxidation and antioxidant enzymes. I review here the most recent data available on the roles of AMPK in vertebrate spermatozoa functions.

## Introduction

One of the fundamental requirements of all cells is to balance ATP consumption and ATP generation. In this regard, ATP hydrolysis is the major source of cellular energy and the study of the involvement of specific molecular mechanisms that modulate its availability is required to understand it better.

In the early 1970s, two groups postulated the existence of a regulator sensitive to the concentration of cytosolic AMP exhibiting an inhibitory effect on molecules linked to the synthesis of fatty acids: Acetyl-CoA carboxylase (ACC) (Carlson and Kim, 1973) and 3-hydroxy-3-methylglutaryl coenzyme A (HMG-CoA) reductase (Beg et al., 1973). In the late 1980s, the implication of a single inhibitor of both ACC and HMG-CoA reductase, still in response to an increase in AMP concentration, was demonstrated (Carling et al., 1987). This discovery has made possible to identify this inhibitory protein as “AMP-activated protein kinase” (Carling and Hardie, 1989). Subsequent research showed that AMPK is concerned with the synthesis of fatty acids, but also with many other metabolic pathways, such as protein or carbohydrate synthesis, enabling it to play not only a key role in the intracellular detection of ATP, but also to have a more global role in maintaining energy balance in cells. From a broader point of view, AMPK was found to act as an energy regulator at both the cellular and the whole-body levels (Carling, 2004).

Recent investigation has elucidated novel biological actions of AMPK that are highly relevant to male reproduction. With the advent of new pharmacological approaches to activate AMPK in a specific manner, the possibility of targeting it in order to improve semen quality is now very close. The focus of the present review is to examine the evidences concerning the actions of AMPK in germ cells, providing new insights into the regulation of AMPK and its biological actions in male reproduction.

## Structure and expression of AMPK

AMPK is a heterotrimeric serine/threonine protein kinase consisting of one catalytic subunit (α) and two regulatory subunits (β and γ) that exist as multiple isoforms and splice variants, resulting in the generation of twelve possible heterotrimeric combinations. The well-conserved genes encoding these subunits are found in the genomes of essentially all eukaryotes, including protists, fungi, plants, and animals (Hardie et al., 2003). AMPK regulates metabolic energy balance at the whole-body level by responding to hormones and nutrient signals, which leads to changes in energy homeostasis (Hardie and Ashford, 2014). Once activated by falling energy status, it promotes ATP production by increasing the activity or expression of proteins involved in catabolism (for example, glucose uptake, glycolysis, fatty acid oxidation and mitochondrial biogenesis). It does so while conserving ATP by switching off biosynthetic pathways such as the synthesis of fatty acids, triglyceride, cholesterol, glucose (via gluconeogenesis) or glycogen (Hardie, 2011).

The α subunit contains a conventional kinase domain containing the threonine-172 (Thr172) which is the major site phosphorylated during activation of AMPK at the N-terminus and a complex with the β and γ subunits at the C-terminal regions (Crute et al., 1998). The β subunit contains two conserved regions: a CBM (carbohydrate-binding module) that is believed to bind to glycogen particles at the N-terminus (Hudson et al., 2003; Polekhina et al., 2003), and a bridge between the α and γ subunits at the C-terminal (Jiang and Carlson, 1997; Thornton et al., 1998; Cheung et al., 2000). The γ subunit carries four cystathionine-β-synthase (CBS) domains repeats which form 2 Bateman domains and is the major binding site of AMP (Bateman, 1997; Adams et al., 2004; Scott et al., 2004). AMP, ADP, or ATP can bind the AMPK γ subunit to regulate AMPK activation through allosteric structural changes in the catalytic α subunit, modulating phosphorylation by upstream kinases, including STK11: serine/threonine kinase 11 [LKB1] (Hawley et al., 2003; Woods et al., 2003), calmodulin-dependent kinase kinase-β [CaMKKβ] (Woods et al., 2005), or TGF-β-activated kinase-1 [TAK1] (Momcilovic et al., 2006; Carling et al., 2008), and regulating the dephosphorylation of Thr172 via phosphatases (phosphatase-2 [PP2A or PP2C]) (Davies et al., 1995).

In addition to the phosphorylation of Thr172 by AMPK kinases, AMPK can be activated, more or less directly, by several pharmacological agents. Metformin and other biguanides, like phenformin (Dykens et al., 2008), activate AMPK by inhibiting Complex 1 of the respiratory chain, which, in turn, decreases intracellular ATP levels (Hardie, 2006; Hawley et al., 2010). Structurally similar to adenosine, AMPK agonist 5-aminoimidazole-4-carboxamide-1-b-d-ribofuranoside (AICAR) is converted by adenosine kinase to the monophosphorylated nucleotide ZMP. ZMP binds to cystathionine β-synthase (CBS) domains of AMPK γ subunit, allowing allosteric activation and increased phosphorylation of Thr172 (Corton et al., 1995). Moreover, some natural compounds, like resveratrol, which is found, for instance, in the skin of red grapes (Baur et al., 2006; Zang et al., 2006), activate AMPK and have a beneficial effect on metabolic diseases comparable to those of AICAR and metformin. This compound acts in a quick fashion by inhibiting the F1F0 mitochondrial ATPase (Hawley et al., 2010) and seems to activate Sirtuins (SIRT1) through increased NAD^+^ levels, induced themselves by AMPK activity (Canto and Auwerx, 2010; Um et al., 2010). Unlike AICAR and metformin, A-769662, a new activator of AMPK (Scott et al., 2014), appears to use a different molecular mechanism to activate AMPK. As it does not increase cellular ADP/ATP or AMP/ATP ratios, A-769662 is considered to directly activate AMPK in cells, expressing an AMP-insensitive mutant (Hawley et al., 2010). Moreover, it does not displace AMP from its binding sites on the γ subunit, suggesting that it binds at a site different from AMP even though, like AMP, it causes both allosteric activation and protection against Thr172 dephosphorylation (Cool et al., 2006; Goransson et al., 2007; Sanders et al., 2007; Scott et al., 2008). A-769662 is also selective for activation of β1 rather than β2 complexes, and its effects are abolished by an S108 mutation in β1 that prevents the autophosphorylation of that serine residue, suggesting that the binding site involved the β subunit (Sanders et al., 2007; Scott et al., 2008, 2014). The activity of AMPK is inhibited by (6-[4-(2-piperidin-1-yl-ethoxy)-phenyl)]3-pyridin-4-yl-pyrazolo[1,5-a] pyrimidine (Zhou et al., 2001) known as compound C or dorsomorphin. It can also block the uptake of AICAR into cells and thus inhibit AICAR stimulatory effects (Fryer et al., 2002). In addition, when incubated with either AICAR or metformin, compound C hinders the inactivation of acetyl CoA carboxylase (ACC) (King et al., 2006).

Among the signaling proteins having a strong impact on the regulation of the functions of somatic and germ cells, AMPK is a crucial cellular energy sensor. Relationships between metabolism and fertility are well established, particularly in the female. Recent discoveries show that AMPK is also present in male gonad and spermatozoa in different species [Caenorhabditis elegans (Lee et al., 2008), oyster (Guevelou et al., 2013), chicken (Nguyen et al., 2014), boar (Hurtado de Llera et al., 2012, 2013), mouse (Tanwar et al., 2012; Tartarin et al., 2012), stallion (Cordova et al., 2014), and human Calle-Guisado et al., 2016; Shabani Nashtaei et al., 2016]. It is these roles in male gonad and spermatozoa that I will now describe.

## Role of AMPK in male gonad and sperm production

The male reproductive system in mammals consists of the testis and a series of ducts and glands. These organs produce semen, which contains spermatozoa as well as other components. Spermatozoa differentiate from germ stem cells in the testis and are transported along the reproductive ducts: the epididymes, the vas deferens, the ejaculatory duct and the urethra (Caroppo, 2011). Testis not only produce gametes (spermatogenesis), but also steroid hormones (steroidogenesis) (Rato et al., 2012; Alves et al., 2013) that control the physiological and behavioral characteristics of male reproduction. Sertoli cells play pivotal roles in 1/ the production and differentiation of germ cells as well as in 2/ estrogen and specific proteins synthesis (ABP (androgen-binding protein), SCF (stem cell factor), AMH (anti-Mullerian hormone), etc.).

AMPK has been detected for the first time in male germinal cells in 2000, and α1 and γ1 isoforms at a higher level than α2 or γ3 isoforms in rat testis (Cheung et al., 2000). AMPK activity in male germinal cells was then presented in 2008 by Towler et al. The kinase upstream of AMPK, called LKB1s, plays a crucial role in male fertility in mice (Towler et al., 2008). Indeed, the absence of LKB1 [LKB1 knockout (KO)] and AMPKα1 [AMPKα1KO] genes in mice decreases spermatozoa production, leading to a reduction of mature spermatozoa in the epididymis in parallel with an increase of the amount of morphologically abnormal, non-motile spermatozoa (Towler et al., 2008), as well as a reduction in mitochondrial membrane potential, a lower basal oxygen consumption, and a decrease in spermatozoa motility (Tartarin et al., 2012). The AMPK α1 subunit is also localized in mouse Sertoli cells (Bertoldo et al., 2016) where the AMPK/mTOR signaling pathway is involved in the regulation of autophagy and apoptosis induced by toxic substances in Sertoli cells (Duan et al., 2016). AMPK activation leads to an increase of lactate production, in response to an increase in glucose uptake and accordingly to increased levels of GLUT1 (glucose transporter 1) and MCT4 (monocarboxylate transporter 4) (Galardo et al., 2007; Um et al., 2010). Specific deletion of the AMPKα1 gene in the Sertoli cells in mice (Sc-AMPKα1KO mice) has led to a 25% reduction in male fertility, associated with abnormal spermatozoa with a thin head and dysregulated energy metabolism with altered lactate, lipid, and ATP production (Bertoldo et al., 2016). In rat Leydig cells, the use of the AMPK activator resveratrol decreased testosterone secretion through a reduction in cholesterol transport into the mitochondria and decreased conversion of progesterone into androstenedione (Svechnikov et al., 2009). In chicken testis, the AMPKα1/2 subunit is found in seminiferous tubules and in Leydig cells, AMPKβ1/2 and AMPKγ1 subunits in seminiferous tubules as well, and AMPKγ3 in Leydig cells (Faure et al., 2016). In chicken (7–10 weeks old), metformin induced a decrease in testis weight, in the proliferative activity of Sertoli cells and in germ cells population, but not in spermatogonia population. Metformin also decreased testosterone levels in chicken Sertoli cells (Faure et al., 2016). These data suggest that the AMPK pathway is involved in the nutritional modulation of the male gonad and spermatozoa production.

Furthermore, the presence of AMPK, especially in its active form, phospho-AMPK-Thr172, was highlighted in spermatozoa of boar, stallion, mouse, chicken and human (Hurtado de Llera et al., 2012, 2013; Tartarin et al., 2012; Cordova et al., 2014; Nguyen et al., 2014; Calle-Guisado et al., 2016; Shabani Nashtaei et al., 2016). In boar, the phospho-AMPK form is mainly localized in the acrosome, the sub-equatorial segment of the head and in the intermediate part (Hurtado de Llera et al., 2013). In stallion, phospho-AMPK has been located in the main piece of the flagellum and the sub-equatorial region of the head (Cordova et al., 2014). In human, AMPK protein is localized all along the spermatozoa, in the entire acrosome, the midpiece, and along the tail of the flagellum (Calle-Guisado et al., 2016; Shabani Nashtaei et al., 2016). In contrast, phospho-AMPK is localized only at the most apical part of the acrosome region and at the spermatozoa tail, and lightly in the post-acrosomal region of the head and in the midpiece region of the tail (Calle-Guisado et al., 2016). In chicken, AMPK is mainly localized in the acrosome, the intermediate part, and the whole flagellum, but phospho-AMPK is especially present in the flagellum and the acrosome, and with a much lower intensity in the intermediate part (Nguyen et al., 2014). The localization of phospho-AMPK in spermatozoa flagellum of several species suggests that it acts through the phosphorylation of protein substrates involved in the functioning of the axoneme central apparatus that is essential for flagellar motility. The mechanism could be similar to that demonstrated for a testicular AMPK-related kinase: TSSK2 (Testis-Specific Serine Kinase 2), a Ser/Thr protein kinase close to AMPK (Scott et al., 2008). *In vitro*, TSKK2 phosphorylates SPAG16L, a protein of the axoneme central apparatus which is essential for mouse spermatozoa flagellar motility (Moreno et al., 2008). By contrast, in chicken, phospho-AMPK-Thr-172 is mainly present in the flagellum and the acrosome, and much less in the intermediate part (Nguyen et al., 2014). These results are in agreement with the fact that the ATP production is not limited to the spermatozoa intermediate piece (mitochondrial respiration) but may also occur at other places via anaerobic glycolysis (Zhou et al., 2001; Scott et al., 2014). Therefore, the AMPK distribution profile in spermatozoa cells suggests that, depending on the species, it promotes in different ways both motility and acrosome reaction. The links between differences in subcellular localization of AMPK and spermatozoa biological characteristics of different species, however, have not yet been defined. In invertebrates, AMPK has also been highlighted in 2013 in oyster's male and female germ cells, where it is involved in the glucose and fatty acids metabolism (Guevelou et al., 2013).

## Role of AMPK in spermatozoa functions

The key role of AMPK in the control of cell energy homeostasis has placed it in a position of an important kinase regarding the regulation of those spermatozoa functions. Indeed, these functions, such as motility, acrosome reaction, and fertilization, are very dependent on energy levels (Hurtado de Llera et al., 2012, 2013; Tartarin et al., 2012; Cordova et al., 2014; Nguyen et al., 2014, 2015; Calle-Guisado et al., 2016; Shabani Nashtaei et al., 2016). AMPK activation in spermatozoa by pharmacological factors favors motility (Nguyen et al., 2014, 2015; Hurtado de Llera et al., 2015; Shabani Nashtaei et al., 2016), acrosome reaction and spermatozoa ability to successfully fertilize the oocyte (Nguyen et al., 2014, 2015). AMPK is also implicated in the mitochondrial membrane potential, spermatozoa plasma membrane fluidity and organization, and acrosome integrity (Hurtado de Llera et al., 2013; Shabani Nashtaei et al., 2016). By contrast, pharmacological inhibition of AMPK, for example with compound C, leads to a reduction in motility parameters such as the percentages of motile and rapid spermatozoa or their curvilinear velocity and average path velocity, in boar, chicken, and human spermatozoa (Hurtado de Llera et al., 2012; Nguyen et al., 2014, 2015; Calle-Guisado et al., 2016; Shabani Nashtaei et al., 2016). Unexpectedly, compound C does not affect spermatozoa functions in stallion (Cordova et al., 2014).

Different AMPK activators exhibit various effects on spermatozoa functions. Metformin promotes no deleterious effect in mouse spermatozoa quality, viability, or integrity of the acrosome membrane, except for a slight reduction in spermatozoa motility when used at high concentration (5 mM), due to a reduction of active mitochondria (Bertoldo et al., 2014). When mouse semen is cryopreserved, spermatozoa viability and membrane integrity is increased by pre-incubating it with metformin before freezing. This also significantly increases the percentage of fertilized oocytes (Bertoldo et al., 2014). Using fresh or frozen-thawed chicken spermatozoa demonstrated that exposure to metformin significantly improves AMPK phosphorylation, spermatozoa motility, viability, and percentage of spermatozoa able to undergo a successful acrosome reaction (Nguyen et al., 2015). The use of metformin in cryopreservation protocols actually seems to have an overall beneficial effect on the quality of cryopreserved semen, improving its quality and fertilization ability, in response to the activation of the AMPK pathway. The other AMPK activators, AICAR and A-769662, exhibit the same beneficial effects as metformin. AICAR increases chicken spermatozoa quality (motility, viability and acrosome reaction) (Nguyen et al., 2014, 2015), and A-769662 improves boar spermatozoa motility (Hurtado de Llera et al., 2015). Supporting a relevant role of AMPK in spermatozoa functions, Tartarin et al. have shown a significant reduction in the fertilizing capacity in mouse epididymal spermatozoa lacking the AMPK catalytic subunit α1 gene [AMPKα1 KO]. This is due to asthenozoospermia and higher rate of morphological and functional abnormalities linked to mitochondrial dysgenesis. There is, however, no changes in testis morphology or spermatozoa production rate. Furthermore, the increase in intra-testicular cholesterol and testosterone, in parallel with a decrease in LH and FSH levels in KO mice, show that the lack of AMPK activation seems to result in an increase of androgens levels, which can negatively influence spermatozoa quality, and consequently, male fertility (Tartarin et al., 2012). Regarding human germ cells, it has been recently shown that resveratrol could activate and phosphorylate AMPK in frozen–thawed spermatozoa, and thus help in restoring their functions, and that compound C had opposite effects (Shabani Nashtaei et al., 2016).

## Role of AMPK in lipid peroxidation and antioxidant enzymes

Oxygen-free radicals, also known as reactive oxygen species (ROS), can be either harmful or beneficial. They are needed for the maturation of cellular structures and help the defense system, supporting cell proliferation and survival pathways (Sundaresan et al., 1995; Echtay et al., 2002; Pacher et al., 2007). But if they are produced in excess, they can damage the cells components and hamper physiological functions (Droge, 2002; Fulda et al., 2010; Kong and Lin, 2010; Martin, 2010; Navarro and Boveris, 2010).

In male reproduction, it has been observed that ROS can disrupt the spermatozoa response to calcium signaling and inhibit the ionophore-induced sperm-oocyte fusion (Aitken and Clarkson, 1987). This could be the result of decreased plasma membrane fluidity and of membrane-bound enzymes and/or ion channels malfunctions, as a consequence of peroxidation (Aitken et al., 1989). ROS have a negative effect on motility which might be due to the depletion of ATP by H_2_O_2_ inhibiting oxidative phosphorylation and/or glycolysis, and thus hindering ATP restoration (deLamirande and Gagnon, 1992a). This process could happen by decreasing phosphorylation of axonemal proteins involved in spermatozoa motility (de Lamirande and deLamirande and Gagnon, 1992b), by depressing glycolytic flux (Baumber et al., 2000), or through products of lipid peroxidation like malondialdehyde or 4-hydroxynonenol (4HN), inhibiting anaerobic glycolysis, DNA, RNA, and protein synthesis (Comporti, 1989).

AMPK appears to be involved in the mechanisms protecting the cells against toxic effects of ROS. Although there are still unknown factors or difficulties in assessing significance of ROS, the goal of many researches is to prevent or counteract their deleterious effects. In forms of diabetes in which the activity of AMPK is strongly impaired, reactivation of AMPK could counteract the negative effects of oxidative stress linked to this metabolic disease (Ceolotto et al., 2007). Resveratrol, by activating AMPK, reduces superoxide anion and protein carbonyl in type II diabetic mice (Chang et al., 2011), and metformin reduces NAFLD in obese Ob/Ob mice submitted to fat diet (Viollet et al., 2012). It is also possible that the AMPK activation caused by resveratrol helps the mitochondria in its defense against oxidative stress by inhibiting ROS generation and permeability transition; it could do that while playing a part in the downstream inhibitory phosphorylation of GSK3b at the same time (Shin et al., 2009). The positive effect of metformin on steatosis depends on the activation of AMPK secondary to the inhibition of complex I of the respiratory chain and the generated energy deficiency (augmented AMP/ATP ratio). An “antioxidant” effect of AMPK has been shown in different systems, and in particular in tumor cells deficient in LKB1 of lung adenocarcinoma. In this model, AMPK may increase the levels of intracellular NADPH in different ways: by the activation of the oxidation of fatty acids and by the inhibition of fatty acid synthesis to neutralize the cytotoxic ROS (Jeon et al., 2012). The antioxidant defense and glutathione regeneration efficiency is greatly improved by NADPH since it is a very important source of reducing equivalent (Fico et al., 2004). Antioxidant enzymes depending on NADPH, like those of the thioredoxin and glutaredoxin classes, are heavily involved in the maintenance of redox homeostasis due to the regulation of thiol-disulfide exchange (Kalinina et al., 2008; Myers and Myers, 2009). While under oxidative stress conditions, AMPK is activated and helps to increase NADPH production through pentose phosphate pathway by starting the glytolytic flux (Wu and Wei, 2012).

In the male reproductive function, a link between AMPK and antioxidant mechanisms also exists. Thus, in the testis of diabetic and obese rats, the use of metformin induces a reduction in the levels of lipid peroxidation (LPO) accompanied by a stimulation of steroidogenesis, a restoration of spermatogenesis, and an increase in spermatozoa concentration and motility (Fang et al., 2012; Nasrolahi et al., 2013). In recent years, the involvement of AMPK in the regulation of ROS or LPO concentrations and the activity of the main antioxidant enzymes in spermatozoa has been demonstrated (Nguyen et al., 2015). The addition of an AMPK activator, metformin or AICAR, before cryopreservation increases the phosphorylation of AMPK while reducing the production of ROS and LPO in chicken frozen/thawed spermatozoa. At the same time, AICAR and metformin increase the activity of antioxidant enzymes superoxide dismutase (SOD), glutathione peroxidase (GPx) and glutathione reductase (GR) in the cryopreserved semen. It has been observed that human spermatozoa incubated with AMPK activator resveratrol have decreased ROS levels (${\text{O}}_{2}^{.-}$ and H_2_O_2_), while AMPK inhibitor increased them (Shabani Nashtaei et al., 2016). Resveratrol had the same effects on mouse spermatozoa, where a 15 μg/mL dosage significantly decreased ROS (Mojica-Villegas et al., 2014). This shows that AMPK activity is limiting ROS production in endothelial cells (Kim et al., 2008), in mice endothelial progenitor cells (by restoring manganese superoxide dismutase (MnSOD) levels) (Wang et al., 2011), and in cryopreserved chicken spermatozoa (Nguyen et al., 2015). However, the mechanisms by which AMPK activators would act on ROS and antioxidant enzyme systems in spermatozoa are still unclear.

AMPK is known to be involved in the renewal of cellular mitochondrial content by stimulating the degradation of defective mitochondria through ULK1/2 (Unc51-like kinase) on the one hand, and by activating biogenesis new mitochondria via PGC1α on the other hand, and thus, indirectly, on antioxidant systems (Canto et al., 2009; Aquilano et al., 2010). But since AMPK stimulation of the regeneration of NADPH (Williams and Ford, 2004) by the pentose phosphate pathway has been demonstrated in mammalian spermatozoa, it probably contributes to the antioxidant system through stimulation of GPx, and could also, as a result, have a role in this function. AMPK could have a part in the up-regulation of several antioxidant enzymes (Colombo and Moncada, 2009; Calegari et al., 2011). It can directly phosphorylate the forkhead transcription factor (FOXO) to promote its nuclear translocation and the formation of subsequent transcription activation complex (Greer et al., 2007). Activating AMPK–FOXO pathway helps up-regulating the expression of thioredoxin and peroxiredoxin and thus reduces oxidant-induced ROS (Chiribau et al., 2008; Li et al., 2009). Through usage of endothelial cells with silenced AMPK α1 subunit, it has also been shown that AMPK induced the expression of genes playing a role in antioxidant defense (like MnSOD, catalase, γ-glutamylcysteine synthase, or thioredoxin) (Colombo and Moncada, 2009). This procedure goes along with a decreased mitochondrial content and increased ROS, suggesting that AMPKα1 involvement in endothelial cells happens in both mitochondrial content and antioxidant defenses. This method also significantly reduced FOXO3a at the protein and mRNA levels, providing further evidence of its role in AMPK antioxidant defense regulation.

I believe that these data will be useful to develop and improve the methodologies of semen handling and storage. Moreover, the positive effects of AMPK stimulation on the functions of spermatozoa remain after cryopreservation (chicken, mice), which supports the hypothesis of its “protector” role on gametes after exposure to extreme heat and osmotic stress conditions encountered during the freeze/thaw process (Bertoldo et al., 2014; Nguyen et al., 2015).

## Spermatozoa AMPK: a link between energy metabolism and fertility

The spermatozoa energy in the form of ATP is mainly obtained from aerobic metabolic (involving the citric acid cycle and oxidative phosphorylation) and/or anaerobic pathways through glycolysis. The metabolic pathways of spermatozoa are mainly localized in mitochondria (Krebs cycle, oxidative phosphorylation) which are found themselves in the intermediate part, the cytoplasm, the fibrous sheath of the flagellum, and the acrosome (Ferramosca and Zara, 2014). AMPK helps to regulate the mitochondrial membrane potential in spermatozoa of boars (Hurtado de Llera et al., 2013) and mice (Tartarin et al., 2012; Bertoldo et al., 2014). An increase in the production of ATP and lactate in spermatozoa cells was observed after the stimulation of AMPK by pharmacological activators (metformin or AICAR) in chicken (Nguyen et al., 2014, 2015). AICAR also stimulates the production of citrate, an intermediate in the Krebs cycle. In the particular case of cryopreservation, after thawing, many spermatozoa die or are unable to move due to alterations of the flagellum or the intermediate piece, or unable to accomplish the acrosome reaction. All these alterations result in a decreased fertility. In these conditions, spermatozoa require a very large amount of energy to restore their fertilizing capacity. The addition of metformin or AICAR before cryopreservation would restore a portion of the energy needed by stimulating the anaerobic metabolism and, for AICAR, the aerobic metabolism (Nguyen et al., 2015). AMPK is therefore essential to keep spermatozoa at the energy level necessary for the conduct of fertilization, especially in “stressful” conditions.

## Conclusions

**Figure 1:**
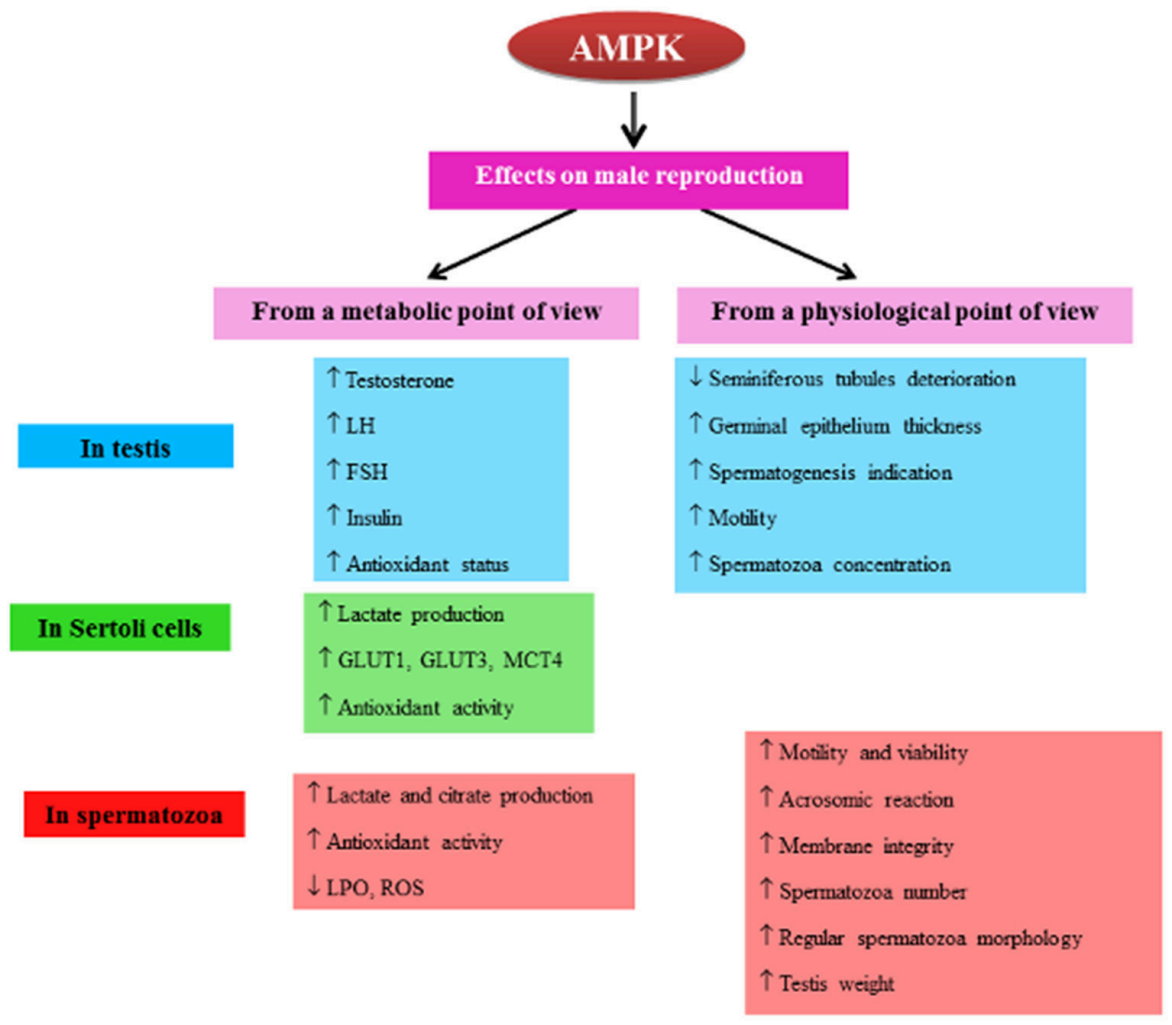
**Diagram of the regulation of AMPK activity in male reproduction**. The diagram represents a summary of AMPK actions in the male reproductive system (testis, Sertoli cells, spermatozoa). T, testosterone; LH, luteinizing hormone; FSH, follicle-stimulating hormone; ABP, androgen-binding protein; SCF, Stem Cell Factor; AMH, anti-Mullerian hormone. GLUT, glucose transporter; MCT, monocarboxylate transporter; ROS, reactive oxygen species; LPO, lipid peroxidation. ↑, increase; ↓, decrease.

All the previously published data and my own results suggest an important role of AMPK in the development of the male gonad and spermatozoa production (Figure 1). It is also involved in spermatozoa functions: in motility and acrosome reaction as well as in the energy and redox capacities in order to regulate cellular processes necessary for successful fertilization. Its role in the regulation of antioxidant defenses of the cell particularly affects the *in vitro* retention capacity, which is especially solicited during the freezing of semen. From a broader point of view, AMPK could become a therapeutic target for limiting infertility and improving biotechnologies of artificial insemination and semen conservation. Further work is needed to study the involvement of AMPK in human male reproductive function.

## Author contributions

TN collected the material and wrote the manuscript.

### Conflict of interest statement

The author declares that the research was conducted in the absence of any commercial or financial relationships that could be construed as a potential conflict of interest.
